# Perceptions of anonymised data use and awareness of the NHS data opt-out amongst patients, carers and healthcare staff

**DOI:** 10.1186/s40900-021-00281-2

**Published:** 2021-06-14

**Authors:** C. Atkin, B. Crosby, K. Dunn, G. Price, E. Marston, C. Crawford, M. O’Hara, C. Morgan, M. Levermore, S. Gallier, S. Modhwadia, J. Attwood, S. Perks, A. K. Denniston, G. Gkoutos, R. Dormer, A. Rosser, A. Ignatowicz, H. Fanning, E. Sapey, On behalf of the PIONEER Data Hub

**Affiliations:** 1 PIONEER Hub in Acute Care, Institute of Inflammation and Ageing, University Hospital Birmingham NHS Foundation Trust, University of Birmingham, Edgbaston, Birmingham, B15 2GW UK; 2 PIONEER HDR-UK Data Hub in Acute Care, Institute of Inflammation and Ageing, University Hospital Birmingham NHS Foundation Trust, University of Birmingham, Edgbaston, Birmingham, B15 2GW UK; 3 HDR-UK Midlands Physical Site, University Hospital Birmingham NHS Foundation Trust, University of Birmingham, Edgbaston, Birmingham, B15 2GW UK; 4 Patient Involvement and Engagement Lead, PIONEER, London, UK; 5 Research Support Services, University of Birmingham, Edgbaston, Birmingham, B15 2TT UK; 6 Research and Development, University Hospital Birmingham NHS Foundation Trust, University of Birmingham, Edgbaston, Birmingham, B15 2GW UK; 7 University Hospital Birmingham NHS Foundation Trust, University of Birmingham, Edgbaston, Birmingham, B15 2GW UK; 8 Public author, B15 2GW Birmingham, UK; 9 Medical Devices Technology International Limited (MDTi), The KaCe Building, Victoria Passage, Wolverhampton, West Midlands WV1 4LG UK; 10 Health, Education and Life Sciences, Birmingham City University, Birmingham, West Midlands UK; 11 Technical Director, PIONEER HDR-UK Data Hub in Acute Care, Institute of Inflammation and Ageing, University Hospital Birmingham NHS Foundation Trust, University of Birmingham, Edgbaston, Birmingham, B15 2GW UK; 12 PIONEER HDR-UK Data Hub in Acute Care, University Hospital Birmingham NHS Foundation Trust, University of Birmingham, Edgbaston, Birmingham, B15 2GW UK; 13 Informatics, University Hospital Birmingham NHS Foundation Trust, University of Birmingham, Edgbaston, Birmingham, B15 2GW UK; 14 Director of INSIGHT - the Health Data Research Hub for Eye Health, University Hospitals Birmingham NHS Foundation Trust, Edgbaston, Birmingham, B15 2GW UK; 15 Centre for Regulatory Science and Innovation, Birmingham Health Partners, Birmingham, B15 2GW UK; 16 NIHR Biomedical Research Centre (Moorfields Eye Hospital NHS Foundation Trust and University College London), Birmingham, UK; 17 Alan Turing Institute, HDR-UK Associated Researcher, Institute of Cancer and Genomic Sciences, University of Birmingham, Edgbaston, Birmingham, B15 2GW UK; 18 Insignia Medical Systems Limited, Paterson House, Hatch Warren Lane, Basingstoke, Hampshire, RG22 4RA UK; 19 West Midlands Ambulance Service Foundation Trust, Millennium Point, Waterfront Business Park, Waterfront Way, Brierley Hill, West Midlands, DY5 1LX UK; 20 Institute of Applied Health Research, University of Birmingham, Edgbaston, Birmingham, B15 2TT UK; 21 Research and Development, University Hospital Birmingham NHS Foundation Trust, University of Birmingham, Edgbaston, Birmingham, B15 2GW UK; 22 PIONEER, HDR-UK Health Data Research Hub in Acute Care, Birmingham Acute Care Research Group, Institute of Inflammation and Ageing, University of Birmingham, Birmingham, B15 2GW UK; 23 Department of Acute Medicine, University Hospitals Birmingham NHS Foundation Trust, Birmingham, B15 2GW UK; 24 NIHR CRF, University Hospitals Birmingham NHS Foundation Trust, Birmingham, B15 2GW UK; 25 PIONEER Health Data Research Hub, University of Birmingham, Edgbaston, Birmingham, B15 2GW UK

**Keywords:** Data sharing, Secondary data use, National Data opt-out, Anonymised healthcare data, Commercial

## Abstract

**Background:**

England operates a National Data Opt-Out (NDOO) for the secondary use of confidential health data for research and planning. We hypothesised that public awareness and support for the secondary use of health data and the NDOO would vary by participant demography and healthcare experience. We explored patient/public awareness and perceptions of secondary data use, grouping potential researchers into National Health Service (NHS), academia or commercial. We assessed awareness of the NDOO system amongst patients, carers, healthcare staff and the public. We co-developed recommendations to consider when sharing unconsented health data for research.

**Methods:**

A patient and public engagement program, co-created and including patient and public workshops, questionnaires and discussion groups regarding anonymised health data use.

**Results:**

There were 350 participants in total. Central concerns for health data use included unauthorised data re-use, the potential for discrimination and data sharing without patient benefit. 94% of respondents were happy for their data to be used for NHS research, 85% for academic research and 68% by health companies, but less than 50% for non-healthcare companies and opinions varied with demography and participant group.

Questionnaires showed that knowledge of the NDOO was low, with 32% of all respondents, 53% of all NHS staff and 29% of all patients aware of the NDOO.

Recommendations to guide unconsented secondary health data use included that health data use should benefit patients; data sharing decisions should involve patients/public. That data should remain in close proximity to health services with the principles of data minimisation applied. Further, that there should be transparency in secondary health data use, including publicly available lists of projects, summaries and benefits. Finally, organisations involved in data access decisions should participate in programmes to increase knowledge of the NDOO, to ensure public members were making informed choices about their own data.

**Conclusion:**

The majority of participants in this study reported that the use of healthcare data for secondary purposes was acceptable when accessed by NHS. Academic and health-focused companies. However, awareness was limited, including of the NDOO. Further development of publicly-agreed recommendations for secondary health data use may improve both awareness and confidence in secondary health data use.

**Supplementary Information:**

The online version contains supplementary material available at 10.1186/s40900-021-00281-2.

## Plain English summary

Health data from routine care can be pseudonymised (with a link remaining to the patient but identifying features removed) or anonymised (with identifying features removed and the link to the patient severed) and used for research and health planning; termed “secondary use”. The National Health Service (NHS) is a single publicly-funded health service for the United Kingdom (UK). The NHS supports secondary data use with a National Data opt-out system. The potential benefits of data secondary use are clear but concerns have been raised. Although the Data Opt-Out is publicised, it is unclear how much public awareness there is of this scheme. We report a patient and publicly created and delivered series of activities including > 350 people; with young adults, patients, NHS staff and the public; to assess concerns, knowledge and acceptance of data sharing.

Perceptions of and support for secondary health data use varied depending on who was asked (by age, gender) and their experience of health services (Staff member, patient, member of the public). Knowledge of schemes to limit secondary data use (such as the UK National Data Op-Out) was low, even among NHS staff. The main concerns of sharing health data included onward data use, the potential for discrimination and exploitation and commercial gain from data use with no benefit to patients. Despite this, most participants agreed with health data sharing with NHS, academic and commercial health-based entities. Agreed, co-created themes to increase the acceptability of health data secondary use included education about ‘Opt-out’ schemes, health service oversight of data use (as the most trusted partner), public and patient involvement in data sharing decisions and public transparency.

**Supplementary Information:**

The online version contains supplementary material available at 10.1186/s40900-021-00281-2.

## Introduction

The National Health Service (NHS) is a single publicly-funded health service for the United Kingdom (UK) which is free at the point of need to the entire population. The NHS necessarily holds identifiable patients’ medical records within specific NHS organisations [1] but this is confidential [2] and classed as sensitive [3]. Electronic health records (EHRs) facilitate data sharing, which is beneficial for the individual [4] especially when care is co-delivered across organisations [5]. Also, EHRs facilitate health data sharing for health service planning, research and innovation, termed secondary use [6] but this usually involves anonymised data [7].

Previous research suggests there is general support for sharing confidential health data for research and planning [8] but there are public concerns [2, 9, 10], especially where data is not fully anonymised or made available for commercial use [8, 11]. This was recently highlighted [12] with 2080 UK citizens who responded to an online survey reporting they were willing to share their data in the following percentages; with academic or medical research institutions (50.3%); a pharmaceutical company (19.8%) or a tech company with an aim to improve health care (12.2%). Little information was provided about the characteristics or healthcare utilisation of the respondents [12] and it is unclear if these preferences might be context dependent – for example people may support the secondary use of health data if people were frequent users of healthcare services or care-givers, or if data sharing was overseen by an organisation local to the participant.

The UK’s NHS is a centralised health system in which the secondary use of data is supported but the capability to opt-out of confidential data use exists, although there is some variation across the UK. In England, organisations can apply to use specific data fields from all patient records, unless patients have ‘opted out’ through the National Data Opt-Out (NDOO) or where there are specific exemptions, such as the recent Control of patient information (COPI) notice for COVID-19 [13, 14]. Where patients have not ‘opted-out’, it is presumed that they have no objection to their data being used for secondary purposes, within the limits of national guidance [2, 14, 15].

Although the NDOO is publicised online and physically in healthcare organisations, studies suggest there is variable public awareness of this scheme [16]. Suggested frameworks to maximise the secondary use of health data often do not include patients’ and citizens’ views [17] although the importance of these views are widely recognised [18]. There is a national consultation to review the Caldicott Principles which guide data use, including consideration of an additional principle that patients’ and service users’ expectations must be considered and informed when confidential information is used [19].

PIONEER is a Health Data Research Hub in acute care, developed to curate routinely-collected health data from unplanned healthcare contacts across community and hospital providers and then facilitate the transparent and ethical use of anonymised data for research and innovation purposes, with a direct aim of improving NHS patient care. PIONEER is based in the West Midlands and to ensure data use reflected the wishes of patients’ whose data are included in the hub, a regional patient and public involvement and engagement (PPIE) programme was initiated, to assess current knowledge and perceptions of health data use.

We hypothesised that public awareness and support for the secondary use of health data and the NDOO would vary by participant demography and healthcare experience. To assess this, we aimed to explore patient/public/carer/NHS staff and NHS volunteer awareness and perceptions of secondary data use, grouping potential ‘data access researchers’ by NHS, academia, healthcare commercial and non-healthcare commercial backgrounds. We aimed to assess the awareness of the NDOO system amongst these groups. Third, we aimed to co-develop with patients, carers and members of the public recommendations to consider when sharing unconsented health data for research.

## Methods

PIONEER is an ethically-approved research database and analytical environment (East Midlands – Derby Research Ethics 20/EM/0158). This PPIE work was conducted following ethical approval from the University of Birmingham Ethical Committee (reference ERN_20–0118).

### Setting and activity

The project included patients and staff from University Hospitals Birmingham NHS Foundation Trust (UHB), one of the largest NHS Trusts in England, with 2750 beds, more than 22,000 staff, a fully EHR (Prescribing Information and Communication System) and a shared primary and secondary care record (Your Care Connected). Members of the public were recruited from public stands and public involvement groups across the West Midlands. For more details of participant recruitment and questionnaire delivery see the online supplement and the individual activities, below.

The project included six activities, running between February 2019 and July 2020, as shown in Table 1.

**Table 1:** The structure of patient and public activity

Activity	Agenda	Objectives	Participants
1. Patient workshop	1. Introduction to health data and electronic health records 2. Health data research (benefits and challenges) 3. Models of data use (Consent versus unconsented, National Data Opt-Out, broad access for hypothesis generating, limited access and the 5 safes) 4. Free discussion – what do attendees think are important considerations in health data use	1. Understand what is meant by health data and how it is stored for primary use 2. Introduce secondary use of health data, de-identification, NHSE opt out and examples of use of health data 3. Agree a list of main concerns about health data use 4. Agree on a list of important themes to be considered in health data use	Patients who had recently been admitted to UHB
2. Public workshop	1. Introduction to health data and electronic health records 2. Health data research (benefits and challenges) 3. Models of data use (Consent versus unconsented, National Data Opt-Out, broad access for hypothesis generating, limited access and the 5 safes) 4. Workshop breakout groups and feedback to all a. Key considerations to using patient data b. What processes should guide the use of patient data c. The use of sensitive data	1. Understand what is meant by health data and how it is stored for primary use 2. Consider examples of how patient data has improved patient care but also risks and challenges 3. Agree a list of main concerns about health data use 4. Agree on a list of important themes to be considered in health data use	Public members from advertisements placed on social media and UHB public forum
3.	Co-creation of questionnaire	Create and test a simple questionnaire to assess knowledge of NHS opt out, and knowledge and perception of health data use.	Patient and public members of PIONEER
4.	Delivery of questionnaire	Delivery of questionnaire to patients, carers, NHS staff and public members including children aged 13 and over	UHB patients, carers and staff. Public members from West Midlands
5. Feedback of questionnaire	Feedback of results of questionnaire to patient working group and public workshop	Assess if wider public consultation reflected the group’s thoughts and where differences were present, why these might be present	Patients and public members in two different sessions

Public members and patients were involved in all stages of the design, delivery, analysis and dissemination of this work.

Participants in the workshops and questionnaire respondents were told that the purpose of the project was to support the development of working practices for PIONEER. Interpreters were used as required.

#### Patient workshop

An open workshop was held with patients of UHB. To be considered a “patient” for this workshop, participants had to self-declare that they had been admitted to UHB hospital within the past year and were members of a patient support group. There were no other inclusion or exclusion criteria. The workshop lasted 4 h with a scribe to take notes. Identified themes were then approved by attendees.

#### Public workshop

To be considered a “public member” for this workshop, participants had to self-declare that they had not been admitted to UHB or any other hospital within the past year. People were asked to self-report their area of residence, age, gender, ethnicity, whether they had been a patient at UHB any other hospital outside of the last 12 months and whether they were a care-giver to people with health challenges (or could choose not report any of these groupings). There were no other inclusion or exclusion criteria. The workshop lasted 4 h with whole group discussion and smaller break out groups. The workshop and break-out groups were audio-recorded with a scribe to take notes. Themes and action points were recorded and then approved by attendees.

#### Questionnaire co-creation and delivery

A short questionnaire was developed by patient/public members and the PIONEER Health Data Research Hub team. See Table S1 of the online supplement for the questions asked. Verbal consent was given by all participants and where children under the age of 18 were approached, their parent, legal guardian or responsible adult provided verbal consent. Participants were asked to self-report their age group, ethnicity, gender, and to place themselves into one of six groups (patient, care-giver, visitor to hospital, NHS staff, NHS volunteer, public member) or they could choose not to report any of these groupings.

### Feedback sessions

Participants at the patient working group and public workshop were invited to a further meeting where the results of the questionnaire were presented and discussed. In a free discussion, participants considered thse results and formed agreed founding principles for data access processes. The workshops were audio-recorded with a scribe to take notes. The identified principles were then approved by attendees.

### Data analysis

Stakeholder workshop audio-recordings were transcribed. Notes of group discussions were also reviewed. As data was collected, thematic analysis was undertaken in an iterative process, searching for commonly expressed views, feelings or words. Summaries and initial themes of the workshops and working groups were shared with participants for their feedback. Participants commented on the findings and particularly on any areas that they felt had been misunderstood. They were also encouraged to make further comments and agree themes.

Demographic data from the questionnaire (age, sex, ethnicity) was compared to the 2011 Birmingham census data and Jan 2019 – Jan 2019 UHB patient episode data. Statistical analyses were performed using IBM SPSS statistics. Comparisons between groups were performed using Chi squared and Fisher Exact tests. A *p* value of < 0.05 was considered statistically significant.

## Results

### Patient workshop

Twelve Birmingham-based patients were included with a median age of 68 years (range 39–78), 58.3% identified as female, the rest as male. They had a median of 3 admissions to hospital in the last year (IQR 2–3.5) and all had chronic respiratory diseases. Five members of the group described themselves of Black, Asian or minority ethnic (BAME) background (41.7%) and seven as of White ethnic group (58.3%).

There was unanimous agreement that routinely collected health data could improve healthcare, with recent examples discussed [20]. The agreed, key concerns about health data use were;

1. Unauthorised data re-use or sharing.
2. Re-identification of the individual
3. Data used to discriminate against groups or the individual.
4. Data being used to generate commercial profit which did not benefit the NHS or UK population.

A key area of discussion was the question of who controlled access to the data. The participants agreed it was important that a trusted partner oversaw data access and use, with the NHS identified as the most trusted partner in the UK. There was agreement that data minimisation was the most appropriate model for access to unconsented health data; with a defined project and data fields, and in accordance with the ‘5 safes’ [21].

83.3% people present had not heard of the National Data opt-out. All thought that data access decisions should be decided in consultation with patients.

The following key points were agreed to help build specific recommendations:

1. Data use was supported if there were benefits to NHS patients or wider population.
2. Most were not aware of the NHSE National Data Opt-Out.
3. Oversight of data use should be provided by the NHS as the most trusted organisation.
4. Unconsented data use should be limited to what is needed;
5. Patient/public involvement in data access decisions.
6. Transparency of data use. There should be an open dialogue with public and patients.

For direct quotes from the workshop, see Table S2 of the online supplement.

### The public workshop

This workshop was attended by 30 delegates. 46.7% were based in Birmingham, 43.3% in the wider West Midlands and 10.0% external to the West Midlands. 46.7% identified as male, 50% as female and 3.3% preferred not to say (0% preferred to self-define gender outside of these groupings). Median age was 52 years (range 23–84) with 20% identifying themselves as from BAME backgrounds. 76.7% had been to an NHS hospital for care previously (but not in the past 12 months and none were undergoing active follow up or treatment). 23.3% self-defined themselves as care-givers for another person with health challenges.

93.3% were happy for health data to be used for research and innovation processes, however all agreed there were risks with health data use

Without knowledge of the patient workshop results, the public workshop agreed the main risks of health data sharing were;

1. Onward data sharing or use without approval
2. Data used against the individual or communities (discrimination and exploitation)
3. Re-identification of the individual
4. Commercial gain from data use (and especially misuse) with no benefit to patients or the UK.

13.3% were concerned about commercial organisations accessing data. All agreed that there should be a searchable record of supported health data access requests. Participants agreed unanimously that patients and the public should be involved in data access processes. Sensitive data or rare conditions were felt more challenging because of the potential consequences or ease of re-identification and that relevant groups should be involved in these data access processes. 76.7% had not heard of the NHS National Data Opt-Out.

The results of the patient workshop were then shared and the group were then asked to consider and agree principles for PIONEER and to form the basis for recommendations to data providers when considering health data secondary use. These were as follows;

1. Health data use with meaningful benefits back to NHS patients and citizens was supported.
2. Data sharing with healthcare providers, academic staff and commercial entities should be considered, as long as there was community support and public awareness campaigns to inform people about how their data was being used.
3. Knowledge of the National Data Opt-Out was low and needed to be improved.
4. Preferably, data should remain in close proximity or within the NHS, with data sharing overseen by the NHS.
5. Data access should be limited to what is needed for a specific project, with agreements about who accesses the data and for how long (in essence, applying the principles of data minimisation).
6. There should be patient/public involvement in data access decisions and key advice sought, especially where sensitive fields or rare conditions were included.
7. There should be transparency in how health data is used, including publicly available lists of projects, summaries and benefits.

For direct quotes from the workshop, see Table S2 of the online supplement.

### Questionnaire results

Demographic details for those that completed the questionnaires are shown in Table 2.

**Table 2:** Demographics of the 308 participants who completed the questionnaires. All category responses were self-defined by respondents

	% (N)	
**Gender**		
Male	39.3%	(121)
Female	58.4%	(180)
Prefer to self-define	0%	(0)
Prefer not to say	2.3%	(7)
**Age**		
13–17 years	19.2%	(59)
18–24 years	5.5%	(17)
25–34 years	17.2%	(53)
35–44 years	12.0%	(37)
45–54 years	13,0%	(40)
55–64 years	13.3%	(41)
65–74 years	13.0%	(40)
75–84 years	5.2%	(16)
85 years or over	1.6%	(5)
**Ethnicity**		
White	70.8%	(218)
Asian/ Asian British	14.6%	(45)
Black/ African/Caribbean/ Black British	7.8%	(24)
Mixed/ multiple ethnic groups	3.2%	(10)
Other	2.9%	(9)
Prefer not to say	0.6%	(2)
**Participant group**		
Patient	22.7%	(70)
Carer	8.4%	(26)
Visitor to hospital	4.9%	(15)
NHS Staff	26.9%	(83)
NHS Volunteer	4.5%	(14)
Public member	28.9%	(89)
Prefer not to say	3.6%	(11)

Demographics of those completing the questionnaire were broadly comparable to patients who used UHB services but there was less representation of Asian participants than the Birmingham catchment area, based on the 2011 census data. See Figure S2 of the online supplement.

#### Current use of anonymised data

**Fig. 1:**
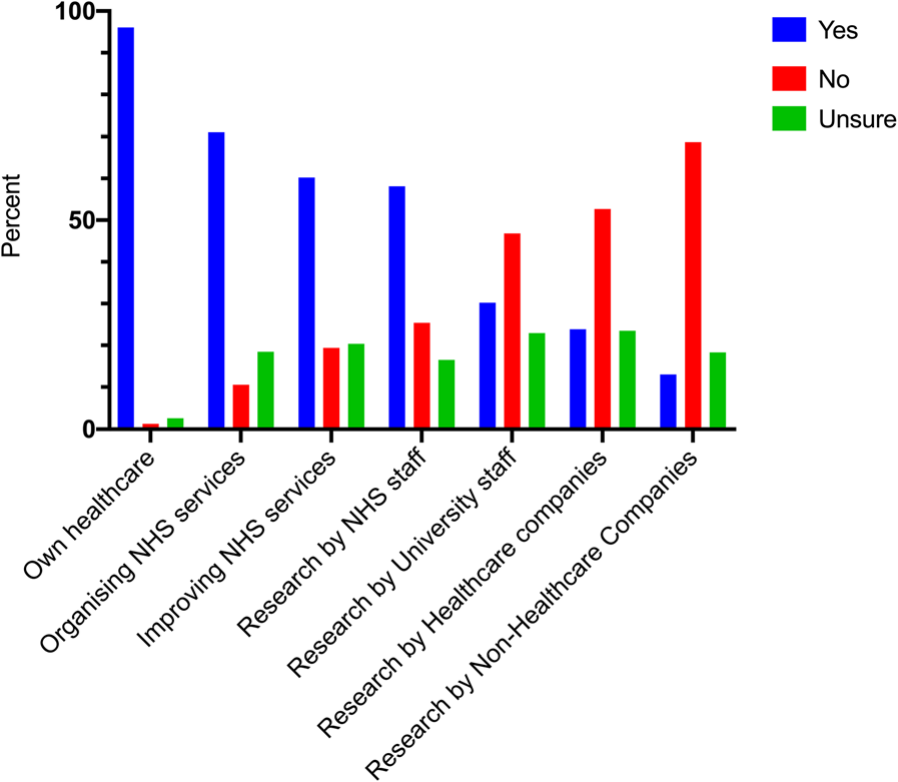
Perception of where healthcare data is currently used. Respondents were asked whether they thought that their health data was currently used for seven purposes, as listed, with a possible answer of yes (shown in grey), no (in black) or unsure (white). All participants answered this question

Figure 1 and Table 3 show how respondents thought their healthcare data was currently used. 96.1% thought their data was used for their own healthcare, 71.0% to improve general NHS services and 60.2% used by research by NHS staff. The majority of participants did not think their health data was used by external agencies including university researchers, or staff from healthcare-focused or non-healthcare focused companies.

**Table 3:** Perception of how anonymised health data is currently used. Respondents were asked whether they believed that their health data was currently used for the seven purposes outlined

	Own healthcare			Organising services within the hospital			Projects which improve NHS services			Research by NHS staff			Research by university researchers			Research by drug companies or medical technology companies			Research by companies who do not provide healthcare products or services		
	Yes	No	Unsure	Yes	No	Unsure	Yes	No	Unsure	Yes	No	Unsure	Yes	No	Unsure	Yes	No	Unsure	Yes	No	Unsure
**Age** (years)																					
12–17	100%	0.0%	0.0%	54.2%	18.6%	27.1%	47.5%	33.9%	18.6%	57.6%	32.2%	10.2%	33.9%	47.5%	18.6%	25.4%	47.5%	27.1%	8.5%	66.1%	25.4%
18–24	100%	0.0%	0.0%	64.7%	5.9%	29.4%	52.9%	29.4%	17.6%	70.6%	17.6%	11.8%	47.1%	41.2%	11.8%	29.4%	58.8%	11.8%	23.5%	64.7%	11.8%
25–34	94.3%	3.8%	1.89%	80.8%	11.5%	7.7%	83.0%	9.4%	7.5%	73.6%	13.2%	13.2%	26.9%	46.2%	26.9%	25.0%	53.8%	21.2%	11.5%	61.5%	26.9%
35–44	94.6%	2.7%	2.7%	67.6%	5.4%	27.0%	54.1%	8.1%	37.8%	48.6%	21.6%	29.7%	18.9%	51.4%	29.7%	21.6%	56.8%	21.6%	10.8%	73.0%	16.2%
45–54	87.2%	2.6%	10.3%	73.7%	10.5%	15.8%	68.4%	10.5%	21.1%	60.5%	13.2%	26.3%	34.2%	31.6%	34.2%	26.3%	31.6%	42.1%	23.7%	52.6%	23.7%
55–64	95.1%	0.0%	4.9%	75.0%	7.5%	17.5%	47.5%	20.0%	32.5%	57.5%	27.5%	15.0%	35.0%	40.0%	25.0%	22.5%	55.0%	22.5%	10.0%	75.0%	15.0%
65–74	100%	0.0%	0.0%	80.0%	12.5%	7.5%	62.5%	25.0%	12.5%	43.6%	43.6%	12.8%	23.1%	64.1%	12.8%	12.8%	71.8%	15.4%	10.3%	87.2%	2.6%
75–84	100%	0.0%	0.0%	73.3%	0.0%	26.7%	66.7%	20.0%	13.3%	53.3%	33.3%	13.3%	35.7%	42.9%	21.4%	40.0%	40.0%	20.0%	14.3%	71.4%	14.3%
85+	100%	0.0%	0.0%	80.0%	0.0%	20.0%	40.0%	20.0%	40.0%	40.0%	40.0%	20.0%	20.0%	80.0%	0.0%	20.0%	80.0%	0.0%	25.0%	75.0%	0.0%
**Gender**																					
Male	96.7%	1.7%	1.7%	74.8%	10.9%	14.3%	63.0%	22.7%	14.3%	61.9%	22.0%	16.1%	34.7%	48.3%	16.9%	25.4%	54.2%	20.3%	16.1%	66.9%	16.9%
Female	96.1%	1.1%	2.8%	67.9%	10.7%	21.4%	59.2%	17.9%	22.9%	56.4%	27.9%	15.6%	27.7%	46.3%	26.0%	22.5%	52.2%	25.3%	10.7%	71.2%	18.1%
**Ethnicity**																					
White	96.3%	1.4%	2.3%	69.6%	10.3%	20.1%	59.1%	20.0%	20.9%	60.3%	22.0%	17.8%	30.5%	44.1%	25.4%	24.4%	49.3%	26.3%	12.7%	67.9%	19.3%
Asian/ Asian British	95.6%	0.0%	4.4%	75.6%	8.9%	15.6%	57.8%	17.8%	24.4%	51.1%	28.9%	20.0%	24.4%	55.6%	20.0%	20.0%	60.0%	20.0%	15.6%	68.9%	15.6%
Black/ African/Caribbean/ Black British	95.8%	0.00%	4.2%	66.7%	16.7%	16.7%	66.7%	20.8%	12.5%	62.5%	33.3%	4.2%	39.1%	52.2%	8.7%	33.3%	58.3%	8.3%	13.0%	69.6%	17.4%
Mixed/ multiple ethnic groups	100.0%	0.00%	0.0%	70.0%	10.0%	20.0%	50.0%	20.0%	30.0%	20.0%	70.0%	10.0%	10.0%	70.0%	20.0%	10.0%	70.0%	20.0%	0.0%	90.0%	10.0%
Other	88.9%	11.1%	0.0%	88.9%	11.1%	0.0%	88.9%	11.1%	0.0%	66.7%	22.2%	11.1%	44.4%	33.3%	22.2%	22.2%	55.6%	22.2%	22.2%	55.6%	22.2%
**Participant group**																					
Patient	100.0%	0.0%	0.0%	77.9%	4.4%	17.6%	57.4%	16.2%	26.5%	48.5%	30.9%	20.6%	30.9%	50.0%	19.1%	23.5%	61.8%	14.7%	14.7%	80.9%	4.4%
Patient carer	96.2%	0.0%	3.8%	76.9%	15.4%	7.7%	46.2%	30.8%	23.1%	42.3%	46.2%	11.5%	30.8%	57.7%	11.5%	19.2%	57.7%	23.1%	16.0%	68.0%	16.0%
Visiting someone	93.3%	0.0%	6.7%	66.7%	13.3%	20.0%	60.0%	6.7%	33.3%	60.0%	13.3%	26.7%	26.7%	33.3%	40.0%	26.7%	40.0%	33.3%	0.0%	80.0%	20.0%
NHS Staff	94.0%	2.4%	3.6%	78.0%	11.0%	11.0%	77.1%	12.0%	10.8%	73.5%	18.1%	8.4%	30.5%	48.8%	20.7%	20.7%	57.3%	22.0%	11.0%	69.5%	19.5%
NHS Volunteer	92.3%	0.0%	7.7%	92.3%	7.7%	0.0%	92.3%	7.7%	0.0%	91.7%	8.3%	0.0%	45.5%	36.4%	18.2%	50.0%	33.3%	16.7%	45.5%	45.5%	9.1%
Public member	93.3%	3.3%	3.3%	56.7%	6.7%	36.7%	43.3%	20.0%	36.7%	40.0%	20.0%	40.0%	16.7%	43.3%	40.0%	16.7%	50.0%	33.3%	13.3%	60.0%	26.7%

Comparing the categories of data use, there was no difference in responses by gender or ethnicity. In general, adult volunteers at the hospital thought that health data was currently being used for more purposes than any other group of respondents. Amongst adult respondents, more staff thought data was currently used for research by NHS researchers than those who were patients (73.5% vs 48.5%, *p* < 0.0005).

#### National Data opt-out (NDOO)

**Fig. 2:**
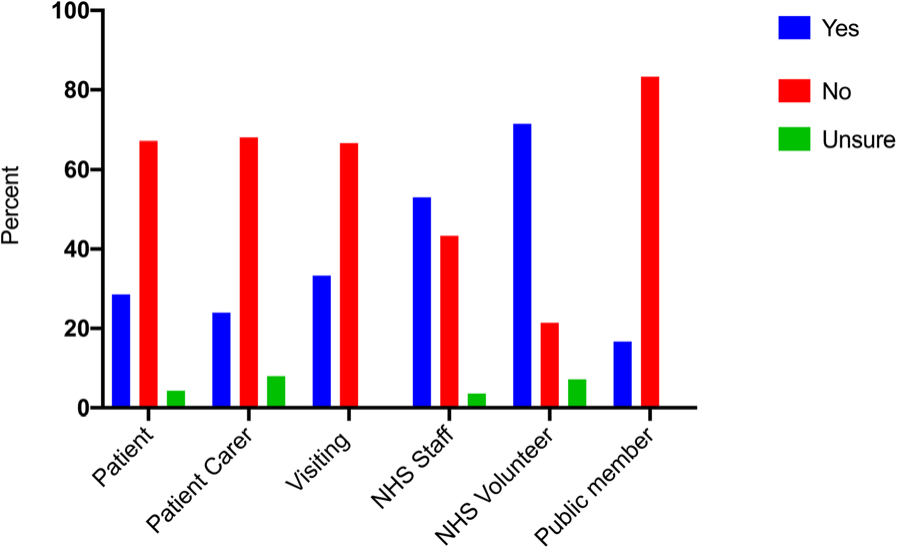
Awareness of NHS data opt-out scheme. Percentage of respondents who were aware of the NHS data opt-out scheme by reason for visiting the hospital or members of the public. All participants answered this question

In total, 31.8% were aware of the NDOO. Table 4 shows the percentage of respondents aware of the Opt-Out within each demographic group. A higher proportion of women than men were aware of the NDOO (36.9% vs 24.2%, *p* = 0.021). A lower proportion of those under 18 years old were aware of the NDOO compared to those who were over 18 years old (10.3% vs 36.8%, *p* < 0.0005). More NHS staff and volunteers were aware of the Opt-Out compared to those with all other groups (*p* < 0.0005) and the groups least aware of the NDOO were members of the public, with less than 20% being aware (Fig. 2).

**Table 4:** Awareness of NHS National Data Opt-Out scheme. Respondents were asked whether they were aware they could opt-out of their anonymised health data being used

	Yes	No	Unsure
**Age** (years)			
13–17	10.3%	84.5%	5.2%
18–24	29.4%	64.7%	5.9%
25–34	44.2%	51.9%	3.9%
35–44	32.4%	64.9%	2.7%
45–54	46.2%	48.7%	5.1%
55–64	22.0%	75.6%	2.4%
65–74	35.0%	57.5%	7.5%
75–84	56.3%	37.5%	6.3%
85+	20.0%	80.0%	0.0%
**Gender**			
Male	24.2%	72.5%	3.3%
Female	36.9%	57.5%	5.6%
**Ethnicity**			
White	33.6%	61.3%	5.1%
Asian/ Asian British	26.7%	71.1%	2.2%
Black/ African/Caribbean/ Black British	41.7%	50.0%	8.3%
Mixed/ multiple ethnic groups	0.0%	100%	0.0%
Other	22.2%	77.8%	0.0%
**Participant group**			
Patient	28.6%	67.1%	4.3%
Patient carer	24.0%	68.0%	8.0%
Visiting someone	33.3%	66.7%	0.0%
NHS Staff	53.0%	43.4%	3.6%
NHS Volunteer	71.4%	21.4%	7.1%
Public member	16.7%	83.3%	0.0%

#### Acceptable data use

**Fig. 3:**
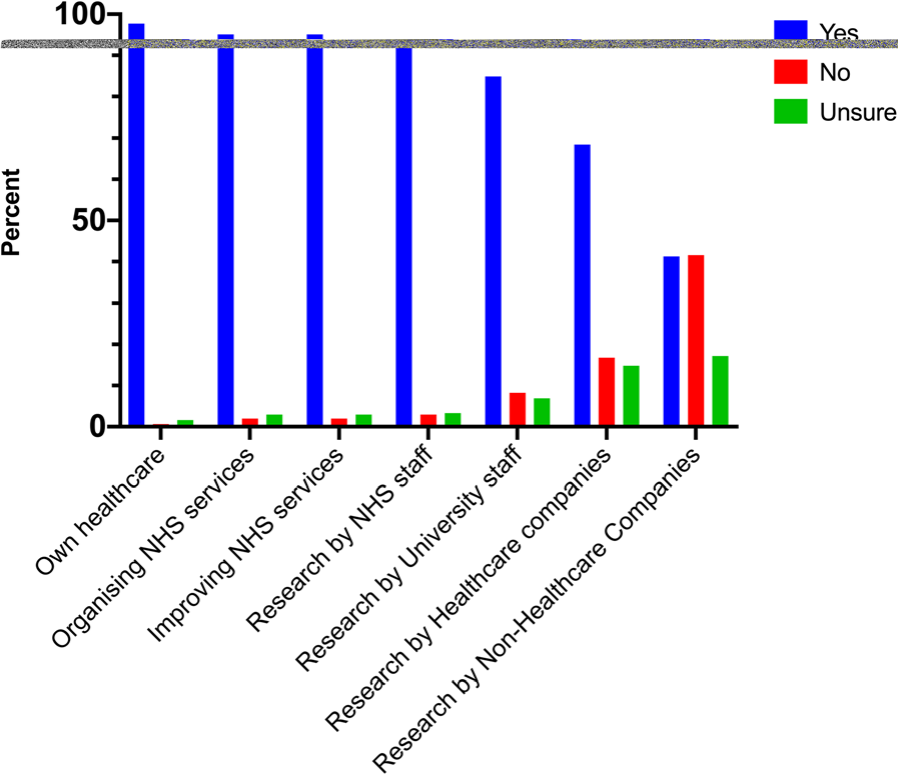
Acceptability of the use of anonymised health data by purpose. Respondents were asked whether they would be happy for their anonymised health data to be used for seven purposes. All participants answered this question

Figure 3 and Table 5 demonstrate the percentage of respondents that would be happy for their anonymised health data to be used for each potential purpose. Three categories were acceptable to more than 90% of respondents: organising NHS services (95.1%); improving NHS services (95.1%); and research by NHS researchers (93.8%). Fewer participants were happy for their data to be used for university researchers than by NHS staff (84.9% vs 93.8%, *p* < 0.0005), although support was high. Over 65% of respondents were happy for their data to be used by healthcare-focused companies but just over 40% were happy for their data to be used by non-healthcare-focused companies. A higher proportion of those aged 12–17 years old were happy for their anonymised data to be used by healthcare-focused companies compared to those aged 65–74 years or 75–84 years (86.4% vs 55 and 33.3%, *p* = 0.001). Less women than men were happy with data use for organising services (92.3% vs 99.2%, *p* = 0.01), projects that improve NHS services (92.8% vs 98.3%, *p* = 0.031), and research by NHS researchers (91.6% vs 97.5%, *p* = 0.038). There were no other significant differences between responses by gender, ethnicity or reason for visit.

**Table 5:** Acceptability of the use of anonymised health data by purpose Respondents were asked whether they would be happy for their anonymised health data to be used for seven purposes, without being asked for their consent

	Own healthcare			Organising services within the hospital			Projects which improve NHS services			Research by NHS staff			Research by university researchers			Research by drug companies or medical technology companies			Research by companies who do not provide healthcare products or services		
	Yes	No	Unsure	Yes	No	Unsure	Yes	No	Unsure	Yes	No	Unsure	Yes	No	Unsure	Yes	No	Unsure	Yes	No	Unsure
**Age** (years)																					
12–17	100.0%	0.0%	0.0%	89.8%	5.1%	5.1%	94.9%	1.7%	3.4%	98.3%	1.7%	0.0%	79.7%	11.9%	8.5%	86.4%	8.5%	5.1%	52.5%	28.8%	18.6%
18–24	100.0%	0.0%	0.0%	94.1%	0.0%	5.9%	94.1%	5.9%	0.0%	93.6%	6.3%	0.0%	82.4%	17.6%	0.0%	82.4%	17.6%	0.0%	64.7%	29.4%	5.9%
25–34	98.1%	0.0%	1.9%	96.2%	1.9%	1.9%	96.2%	1.9%	1.9%	94.3%	1.9%	3.8%	86.8%	9.4%	3.8%	62.3%	26.4%	11.3%	41.5%	54.7%	3.8%
35–44	89.2%	5.4%	5.4%	94.6%	2.7%	2.7%	91.9%	5.4%	2.7%	91.9%	5.4%	2.7%	83.8%	10.8%	5.4%	73.0%	13.5%	13.5%	43.2%	45.9%	10.8%
45–54	97.4%	0.0%	2.6%	97.4%	0.0%	2.6%	97.4%	0.0%	2.6%	92.3%	5.1%	2.6%	76.9%	7.7%	15.4%	61.5%	17.9%	20.5%	35.9%	41.0%	23.1%
55–64	100.0%	0.0%	0.0%	97.5%	2.5%	0.0%	95.0%	2.5%	2.5%	92.5%	2.5%	5.0%	92.5%	2.5%	5.0%	75.0%	10.0%	15.0%	41.0%	46.2%	12.8%
65–74	97.5%	0.0%	2.5%	97.5%	0.0%	2.5%	95.0%	0.0%	5.0%	90.0%	2.5%	7.5%	92.5%	2.5%	5.0%	55.0%	15.0%	30.0%	22.5%	40.0%	37.5%
75–84	100.0%	0.0%	0.0%	93.8%	0.0%	6.3%	93.8%	0.0%	6.3%	93.3%	0.0%	6.7%	80.0%	6.7%	13.3%	33.3%	40.0%	26.7%	26.7%	46.7%	26.7%
85+	100.0%	0.0%	0.0%	100.0%	0.0%	0.0%	100.0%	0.0%	0.0%	100.0%	0.0%	0.0%	100.0%	0.0%	0.0%	50.0%	25.0%	25.0%	50.0%	25.0%	25.0%
**Gender**																					
Male	99.2%	0.0%	0.8%	99.2%	0.8%	0.0%	98.3%	0.8%	0.8%	97.5%	0.8%	1.7%	85.0%	8.3%	6.7%	73.3%	15.8%	10.8%	43.7%	43.7%	12.6%
Female	96.6%	1.1%	2.2%	92.8%	2.8%	4.4%	92.8%	2.8%	4.4%	91.6%	3.9%	4.5%	86.0%	6.7%	7.3%	65.4%	16.8%	17.9%	40.8%	39.1%	20.1%
**Ethnicity**																					
White	97.2%	0.5%	2.3%	94.4%	1.9%	3.7%	94.4%	1.4%	4.2%	94.9%	1.4%	3.7%	83.3%	8.3%	8.3%	69.4%	15.3%	15.3%	39.5%	41.9%	18.6%
Asian/ Asian British	97.8%	2.2%	0.0%	95.6%	4.4%	0.0%	95.6%	4.4%	0.0%	88.9%	8.9%	2.2%	86.7%	8.9%	4.4%	62.2%	22.2%	15.6%	48.9%	42.2%	8.9%
Black/ African/Caribbean/ Black British	100.0%	0.0%	0.0%	100.0%	0.0%	0.0%	100.0%	0.0%	0.0%	91.3%	4.3%	4.3%	87.0%	8.7%	4.3%	56.5%	26.1%	17.4%	34.8%	39.1%	26.1%
Mixed/ multiple ethnic groups	100.0%	0.0%	0.0%	90.0%	0.0%	10.0%	90.0%	10.0%	0.0%	90.0%	10.0%	0.0%	90.0%	10.0%	0.0%	80.0%	10.0%	10.0%	40.0%	50.0%	10.0%
Other	100.0%	0.0%	0.0%	100.0%	0.0%	0.0%	100.0%	0.0%	0.0%	100.0%	0.0%	0.0%	100.0%	0.0%	0.0%	100.0%	0.0%	0.0%	66.7%	22.2%	11.1%
**Participant group**																					
Patient	97.1%	0.0%	2.9%	95.7%	0.0%	4.3%	95.7%	0.0%	4.3%	92.8%	1.4%	5.8%	94.2%	1.4%	4.3%	63.8%	13.0%	23.2%	41.2%	32.4%	26.5%
Patient carer	100.0%	0.0%	0.0%	96.0%	4.0%	0.0%	96.0%	4.0%	0.0%	92.3%	3.8%	3.8%	88.0%	4.0%	8.0%	72.0%	16.0%	12.0%	52.0%	28.0%	20.0%
Visiting someone	100.0%	0.0%	0.0%	100.0%	0.0%	0.0%	100.0%	0.0%	0.0%	93.3%	6.7%	0.0%	93.3%	6.7%	0.0%	86.7%	13.3%	0.0%	13.3%	86.7%	0.0%
Here because of my work	95.2%	2.4%	2.4%	96.4%	2.4%	1.2%	94.0%	3.6%	2.4%	91.6%	4.8%	3.6%	85.5%	8.4%	6.0%	62.7%	25.3%	12.0%	41.0%	51.8%	7.2%
Volunteer	100.0%	0.0%	0.0%	100.0%	0.0%	0.0%	100.0%	0.0%	0.0%	100.0%	0.0%	0.0%	84.6%	15.4%	0.0%	53.8%	30.8%	15.4%	30.8%	53.8%	15.4%
Public member	100.0%	0.0%	0.0%	100.0%	0.0%	0.0%	100.0%	0.0%	0.0%	100.0%	0.0%	0.0%	86.7%	3.3%	10.0%	63.3%	10.0%	26.7%	40.0%	40.0%	20.0%

#### Current use compared to acceptable use

Responses for each category were assessed, comparing how respondents thought data was currently used and whether they would be happy for the data to be used. For all categories, a significantly higher proportion of respondents said they would be happy for their data to be used than thought it was currently used for this purpose (Table 6).

**Table 6:** Comparison of proportion of respondents who think their health data is currently used for each suggested purpose, compared to the proportion that would be happy for their data to be used in this way

	Think is currently used for this reason	Would be happy for use for this reason	*p* value
Own healthcare	96.1%	97.7%	0.244
Organising services within the hospital	71.0%	95.1%	< 0.005
Projects which improve NHS services	60.2%	95.1%	< 0.005
Research by NHS staff	58.1%	93.8%	< 0.005
Research by university researchers	30.2%	84.9%	< 0.005
Research by drug companies or medical technology companies	23.8%	68.4%	< 0.005
Research by companies who do not provide healthcare products or services	13.0%	41.3%	< 0.005

### Feedback groups

The results of the questionnaires were presented and discussed with the patient and public workshop attendees. There was agreement across the attendees that health data use was broadly supported, especially when the researchers were associated with organisations related to healthcare. Also, that as knowledge of the NHS National Data Opt-Out was low, people did not have the opportunity to exercise their right to opt out through a potential lack of awareness. The seven principles to guide unconsented health data use described above were ratified without change and were put forward as recommendations for organisations who are involved in providing access to health data.

## Discussion

Different countries operate different legal frameworks for health data access, with the UK choosing a National Data Opt-Out (NDOO) [14] working within the principles of the General Data Protection Regulation (as defined initially by the European Union [22] and Information Commissioner’s Office [23]. Recent questionnaires suggest variable support for data access, but this may depend on the population asked. Few people have asked young adults and the views of frequent healthcare users or care-givers may differ from those without chronic illnesses. There may be regional differences related to where the data is stored and which organisation is Data Controller. The PIONEER Hub contains health data from the West Midlands and so the opinions of local patients and citizens were sought. We describe the initial phases of a program of work to explore these themes.

In general, there was little knowledge of, but then support for the use of anonymised health data for secondary purposes by NHS, academic and commercial organisations, providing there was a link to healthcare and that there was patient and public involvement and engagement in data-sharing decisions and outcomes. Only research by non-healthcare-focused commercial organisations received less than 50% support. The reported support for health data use in the current paper are much higher than reported recently [12]. The reasons for the disparity are unclear. This might reflect differences in the population questioned, or the inclusion of patients and visitors to hospital and NHS staff. The current study included people who self-selected to take part in this series of workshops or who agreed to answer a questionnaire, and therefore views expressed may not be reflective of the wider population and could form a source of bias including selection and sampling bias. However, the demographics of respondents were broadly similar to the West Midlands population, and information was available for translation as needed. A wider survey of the population would be needed to reduce bias, although even here non-response bias can be difficult to overcome [24]. In the current study, participants were asked to comment on healthcare-focused or non-healthcare-focused companies, but there are a wide variety of healthcare companies and people might feel more comfortable with health data secondary use if different examples of benefit could be clearly provided across this diverse sector.

Awareness of the NDOO system was low including in NHS staff. This suggests that increased education is needed for the public, patients and carers, as well as those working in healthcare. In the patient and public workshops within the current study, it was agreed that knowledge of the NDOO was low and needed to be improved. Currently, training about the secondary use of health data or the NDOO is not taught routinely to healthcare professionals or healthcare students and there are minimal opportunities for patients or public members to be informed about health data use for research and planning. Introducing the benefits that come from health data secondary use and the NDOO within the mandatory training undertaken annually by healthcare staff may be an effective means to increase awareness. Providing more opportunities for non-healthcare professionals to learn about the NDOO through webinars or public events may also increase awareness, with data providers working synergistically.

Previous surveys also suggest that public citizens have greater confidence in data use for research conducted through the NHS than by pharmaceutical companies [25]. In our study, the NHS was consistently identified as the most trusted partner to hold data or make decisions on health data use, mirroring the findings of a recent OneLondon event [26] and previous research [11, 27].

The results from our study are in keeping with those recently published from Understanding Patient Data, which described a majority of people believing the public should be involved in decisions about how NHS data is used and that benefits from health data partnerships should be shared across the NHS [28].

The principles of data minimisation (access only to what data was needed and no more, by only those who needed to access the health data) viewed favourably by our participants has also been highlighted in previous engagement events [26] and may help to increase acceptability of data use for secondary purposes [29].

Those aged 13 years or older were included here as individuals can choose to use the NDOO from the age of 13 (see [30] for more details). Our results suggest those under 18 may be more accepting of anonymised data use by healthcare-focused companies than older adults, and that they had lower awareness of the NDOO that adults (10% aware), but this requires further study. A previous study of the perception of electronic health records found that 60% of young people did not understand how healthcare records could be beneficial to research, which improved after education [31].

This study has limitations. The sample size was limited, especially of patients in the initial workshop and results should be interpreted with this in mind, including the potential for bias. Participants included patients, carers, NHS staff, NHS volunteers and members of the public within the West Midlands, and should not be extrapolated across other populations, nor considered to definitively represent the views of the local or wider population. Further work is needed to understand if perceptions of health data use differ across different groups or communities. Workshops allowed for a more in-depth assessment of patient and public perceptions, but did not fully explore why participants would not be happy for their data to be used for specific purposes.

## Conclusion

This study highlights the continued lack of awareness of the secondary use of health data and the NDOO. There is a clear need to increase awareness of and provide greater clarity about the use of health data for research and planning, including the benefits this can provide to patients and the wider public. Related to this is a need to inform people (including healthcare staff) about the NDOO within the UK, enabling people to make an informed decision about how aspects of their health data are used. Potential means to do this might include information about data use and the NDOO during healthcare student courses or during the annual mandatory healthcare training that all healthcare professionals must attend. For patients, information could be provided during admissions to hospital or out-patient attendances. For members of the public, a series of public events, including webinars, leaflets and meetings, may enhance public knowledge. Developing and testing the most effective means to enhance knowledge requires further research.

Patients and members of the public co-developed a series of recommendations for data-providers to consider when engaging in data sharing activities, which included considering patient benefit, transparency and patient/public involvement, proximity to healthcare services and data minimisation. These require ratification by different public groups and further research to test if the implementation of these recommendations reduces concerns or increases public confidence in data sharing. However, these recommendations are concordant with individual outputs made by other public workshops, and were unanimously agreed by patients, care-givers and members of the public, suggesting they should be considered in data sharing activities.

## Supplementary Information

**Additional file 1: Table S1.** Questions asked regarding data use. **Table S2.** Direct quotes from the workshops. **Figure S1.** Demographics of questionnaire respondents, University Hospitals Birmingham NHS Foundation Trust patients and population of Birmingham. a) Ethnicity; b) age for adults aged 25 years and over. Birmingham data was based on the 2011 census data. Hospital patients were based on all patient episodes from January 2019 to January 2020 (data from PIONEER Hub). There was less representation of Asian/ Asian British ethnicity in the questionnaire than in the Birmingham catchment area (*p* = 0.004), but no differences in other ethnic groups and no differences in ethnicity groups from UHB patients (all episodes January 2019 – January 2020). There were more people aged 65 – 74 in the questionnaire than the Birmingham catchment area census data, but no other significant differences in age from the Birmingham census data.

**Additional file 2.**

## Data Availability

All structured data is available from the PIONEER Hub upon reasonable request. Please contact the corresponding author for details.

## Abbreviations

BAME: Black, Asian, Minority Ethnic
EHR: Electronic Health Record
HDR-UK: Health Data Research - UK
NDOO: National Data Opt-Out
NHS: National Health Service
NHSE: National Health Service England
PPIE: Patient/Public Involvement/ Engagement
UHB: University Hospital Birmingham NHS Foundation Trust
UK: United Kingdom
