# Supplementary material for: Perceptions of anonymised data use and awareness of the NHS data opt-out amongst patients, carers and healthcare staff
Source: Res Involv Engagem. 2021 Jun 14;7:40. doi: 10.1186/s40900-021-00281-2 (PMC8201435; doi:10.1186/s40900-021-00281-2)
Supplement: Supplementary file 1 — Additional file 1: Table S1. Questions asked regarding data use. Table S2. Direct quotes from the workshops. Figure S1. Demographics of questionnaire respondents, University Hospitals Birmingham NHS Foundation Trust patients and population of Birmingham. a) Ethnicity; b) age for adults aged 25 years and over. Birmingham data was based on the 2011 census data. Hospital patients were based on all patient episodes from January 2019 to January 2020 (data from PIONEER Hub). There was less representation of Asian/ Asian British ethnicity in the questionnaire than in the Birmingham catchment area (p = 0.004), but no differences in other ethnic groups and no differences in ethnicity groups from UHB patients (all episodes January 2019 – January 2020). There were more people aged 65 – 74 in the questionnaire than the Birmingham catchment area census data, but no other significant differences in age from the Birmingham census data. [file 40900_2021_281_MOESM1_ESM.docx]

**Online supplement: Perceptions of anonymised data use and awareness of the NHS data opt-out amongst patients, carers and healthcare staff**

**Methods**

**Participant selection for activities**

Patient workshops were from pre-formed community patient groups, and all attendees were asked to take part.

Public workshops were advertised locally both through posters on community boards. Potential participants had to register to attend. A maximum of 32 participants was agreed (with two participants dropping out on the day). There were no pre-participation selection processes, but the posters requested that people were not actively under UHB hospital for care at the time of the workshops or had been admitted to hospital in the past twelve months.

Inclusion criteria are given in the main article.

For questionnaires, stalls were set up in the main entrance of the QEH hospital Birmingham, and all potential participants who passed the stall were asked to participate sequentially, with no exceptions. For public opinions, stalls were set out in local community facilities including sports clubs and community centres. Again, all who passed the stall were asked if they would like to participate. Data collection ran over 3 days, for a two-hour period each time.

All workshops were run by members of the University of Birmingham in plain clothes.

Table S1: Questions asked regarding data use.

Question 1: What do you know about how your health data is currently used?

Do you think that your health data is used for? (answer ‘yes’, ‘no’ or ‘unsure’)

- Your own healthcare
- Organising services within the hospital (surgery lists, clinic times)
- Projects which improve NHS services for all patients
- Research undertaken by NHS staff
- Research undertaken by University researchers who are not linked to the NHS
- Research by drug companies or medical technology companies
- Research by companies who do not provide healthcare products or services

Question 2: Your health data can be used (in an anonymised form, so no one can identify you) for all of these reasons. Unless you “opt out” your health data can be used to help the NHS improve the care it provides to you and others. Did you know that you could “opt out” of your health data being used in this way? (answer ‘yes’, ‘no’, or ‘unsure’)

Question 3: In general, are you happy about your anonymised health data (so no one knows it is you) being used for the reasons given below;

I would be happy for my anonymised health data to be used for (answer ‘yes’, ‘no’ or ‘unsure’)

- Your own healthcare
- Organising services within the hospital (surgery lists, clinic times)
- Projects which improve NHS services for all patients
- Research undertaken by NHS staff
- Research undertaken by University researchers who are not linked to the NHS
- Research by drug companies or medical technology companies
- Research by companies who do not provide healthcare products or services

| **Discussion Theme** | **Patient workshop** | **Public workshop** |
| --- | --- | --- |
| Benefits | *“I can see very how joined up health information will help doctors make better decisions for their patients”* Ms X (54 yrs)  *“I am happy to share my health data if it helps anyone else” Ms X (72yrs)*  *“Sometimes you learn much later about a rare problem that was not picked up before”* Mr X (71yrs) | *“Everyone is included, so it has to help make better decisions”* Ms Y (45yrs)  *“You can see the progress and improvements since health records were digital*” Mr Y (54yrs) |
| Concerns | “*It doesn’t take much to identify a person and that is a concern”* Mr X (67yrs)  *“I worry who else will see that information, and how they will use it”* Mr X (39yrs)  “*What if that data was used by insurance companies or banks to make decisions about people*” Mr X (48yrs)  *“You hear about large companies buying data for a pittance and then making a huge profit”.* Mr X (71yrs)  *“How can we make sure the NHS benefits – it is our records after all”* Ms X (54yrs*)* | *“You need to protect against people knowing who the person is from the data shared” Mr Y (35 yrs)*  “*Can you know what other information they have, how long they have been holding it for and what else they are going to do with it?”* Ms Y (64yrs)  *“Companies will use that information to cut their costs, perhaps by stopping some services for some people”* Mr Y (51 yrs)  “*Sharing health records with companies and foreign governments is a big concern – the NHS may not see a penny of that back*” Mr Y (69 yrs) |
| Principles to guide health data use | “*The most important thing is that the data helps people, especially patients*” Mr X (71 yrs)  “*It makes sense to share information with anyone who can use it to help treat patients, but there have (sic) to be clear rules about how that information is used and who by*” Mr X (48 yrs)  “*People need to know how health information is used, and how they can opt-out if they want to”* Mr X (70 yrs)  “*It is more reassuring if the NHS held the data and were involved throughout”* Ms X (69 yrs)  “*There needs to be more involvement of patients in these decisions”* Mr X (39 yrs)  *“A list of projects that health data has supported and what these projects delivered – spread the good news”* Mr X (48 yrs) | “*Any improvements to health care made from our data should benefit the NHS or our communities”.* Mr Y (70yrs)  “Companies should be able to access health data, but patients should know about this and there need to be limits in what they can see and what they can use it for” Ms Y (23 yrs)  “We need to tell more people about the Opt-out. It is not a choice if you don’t know about it” Ms Y (66 yrs)  “Who gets to decide on data sharing? It should be a balance of experts, doctors and members of the public” Mr Y (53 yrs)  “Patients with the disease should have a say in how their information is used” Ms Y (49yrs)  “NHS staff are probably the best people to mind our data. They have training and experience in this” Mr Y (35 yrs)  “More involvement in these decisions will improve public trust”. Ms Y (64 yrs) |

Table S2. Direct quotes from the workshops.

a)

b)

Figure S1: Demographics of questionnaire respondents, University Hospitals Birmingham NHS Foundation Trust patients and population of Birmingham. a) Ethnicity; b) age for adults aged 25 years and over. Birmingham data was based on the 2011 census data. Hospital patients were based on all patient episodes from January 2019 to January 2020 (data from PIONEER Hub). There was less representation of Asian/ Asian British ethnicity in the questionnaire than in the Birmingham catchment area (p = 0.004), but no differences in other ethnic groups and no differences in ethnicity groups from UHB patients (all episodes January 2019 – January 2020). There were more people aged 65 – 74 in the questionnaire than the Birmingham catchment area census data, but no other significant differences in age from the Birmingham census data.
