# Supplementary material for: Perceptions of anonymised data use and awareness of the NHS data opt-out amongst patients, carers and healthcare staff
Source: Res Involv Engagem. 2021 Jun 14;7:40. doi: 10.1186/s40900-021-00281-2 (PMC8201435; doi:10.1186/s40900-021-00281-2)
Supplement: Supplementary file 2 — Additional file 2. [file 40900_2021_281_MOESM2_ESM.pdf]

GRIPP2 Short Form

**Section and Topic**	**Item**	**Reported on Page Number**
1: Aim	Report the aim of patient and public involvement (PPI) in the study	4
2: Methods	Provide a clear description of the methods used for PPI in the study	5 - 6 and online supplement
3: Study results	Outcomes - Report the results of PPI in the study, including both positive and negative outcomes	7 - 14
4: Discussion and conclusions	Outcomes - Comment on the extent to which PPI influenced the study overall. Describe positive and negative effects	14-15
5: Reflections/critical perspective	Comment critically on the study, reflecting on the things that went well and those that did not, so others can learn from this experience	15
